# Enhancing Salt Tolerance in Tomato Plants Through PEG6000 Seed Priming: Inducing Antioxidant Activity and Mitigating Oxidative Stress

**DOI:** 10.3390/plants14091296

**Published:** 2025-04-25

**Authors:** Nasratullah Habibi, Shafiqullah Aryan, Naveedullah Sediqui, Naoki Terada, Atsushi Sanada, Atsushi Kamata, Kaihei Koshio

**Affiliations:** 1 Graduate School of Agriculture, Tokyo University of Agriculture, 1-1-1 Sakuragaoka, Setagaya-ku, Tokyo 156-8502, Japan; naweedone@gmail.com (N.S.); nt204361@nodai.ac.jp (N.T.); a3sanada@nodai.ac.jp (A.S.); koshio@nodai.ac.jp (K.K.); 2 Faculty of Agriculture, Balkh University, Mazar-e-Sharif 1701, Balkh, Afghanistan; 3 Faculty of Agriculture, Nangarhar University, Jalalabad 2601, Nangarhar, Afghanistan; shafiqaryan@gmail.com; 4 Faculty of Agriculture, Tokyo University of Agriculture, Isehara Farm, 1499-1 Maehata, Sannomiya, Kanagawa, Isehara 259-1103, Japan; ak207913@nodai.ac.jp

**Keywords:** antioxidant activity, fruit quality, salt stress, seed priming, tomato

## Abstract

Salt stress is a major constraint to crop productivity, negatively affecting plant physiology and fruit quality. This study hypothesized that seed priming with polyethylene glycol (PEG6000) might enhance antioxidant activity by mitigating oxidative stress in *Solanum lycopersicum* ‘Micro-Tom’ under salt stress. Seeds primed with –1.2 MPa PEG6000 were grown in Rockwool and treated with 0, 50, 100, 150, and 200 mM NaCl. Primed plants showed a 32% increase in leaf potassium (K^+^) and a 28% decrease in sodium (Na^+^) accumulation compared to non-primed plants under 150 mM NaCl. Glucose, fructose, and sucrose contents increased by 25%, 22%, and 19%, respectively, in primed fruits, while citric acid decreased by 15%. Malondialdehyde (MDA) and electrolyte leakage were reduced by 35% and 29%, respectively, in primed plants under moderate salinity. Antioxidant enzyme activities—SOD, POD, CAT, and APX were enhanced by 30–45% in primed plants under 100 and 150 mM NaCl, compared to non-primed controls. Abscisic acid (ABA) levels increased by 40% in primed roots under salt stress. Activities of polyamine-related enzymes (DAO, PAO, and ADC) also rose significantly. Priming improved protein content by 20% and relative water content by 18%. These results suggest that PEG6000 seed priming enhances salt tolerance by boosting antioxidant defense, regulating osmotic balance, and improving ion homeostasis, offering a viable strategy for sustaining tomato productivity under salinity.

## 1. Introduction

The tomato is a vegetable with immense nutritional benefits [1]. It is one of the most consumable vegetables in the world [2]. However, stress factors significantly decrease tomato yield, and one major factor is soil salinity [3,4]. Salinity emerges as a conspicuous abiotic stressor, casting a substantial shadow of adversity over plant ecosystems [5,6]. This abiotic stress reduces plant growth [7,8,9] and crop yield [10,11]. Salinity disrupts the water balance within plant cells [12,13], leading to a pronounced osmotic stress that hinders normal metabolic functions [14]. Simultaneously, it interferes with nutrient absorption, with high salt concentrations in the soil hampering the plant’s ability to access vital elements [15]. Furthermore, the presence of excess ions, notably sodium and chlorine [11], induces toxicity, perturbing cellular homeostasis. This, in turn, triggers oxidative stress [16], as heightened levels of reactive oxygen species accumulate, subjecting plant cells to damage [17]. Additionally, salinity exerts an inhibitory influence on crucial physiological processes [18,19,20], most notably photosynthesis [21,22]. Hormone regulation, which plays a pivotal role in growth and development, is also disrupted by salinity’s pervasive impact [23], affecting plant vitality [24,25] and fruit quality [26,27,28]. The intricate interplay of these consequences collectively culminates in the stunted growth and decreased productivity of plants in saline environments. Mitigating these multifaceted challenges has become a pressing concern in contemporary agriculture and plant science, emphasizing the need for innovative strategies to enhance salinity tolerance in crops.

Numerous strategies can be harnessed to ameliorate the deleterious impact of salinity on plant growth, among them, seed priming [29,30]. This stands out as a relatively straightforward yet pivotal technique. Seed priming is a controlled hydration technique that initiates early metabolic processes in seeds without allowing radicle emergence. After priming, seeds are re-dried, enhancing germination speed, seedling vigor, and stress tolerance. In contrast, seed pre-treatment broadly refers to any physical, chemical, or biological treatment applied to seeds before sowing, such as fungicide coating or scarification, that may not activate metabolic processes. Thus, seed priming is a physiological activation method, while pre-treatment often involves external modifications [31]. Seed priming entails immersing seeds in a solution [32,33,34,35] to augment their germination and early growth [36,37], especially in saline environments [20]. Primed seeds exhibit accelerated germination rates and require less time for seedling establishment [38]. Furthermore, these primed seeds give rise to superior shoots [38] and root development [39], thereby enhancing their capacity to access water [40] and essential nutrients [41] even in the face of salt-induced stress. As a result, seed priming bolsters salinity tolerance [42,43,44,45], leading to improved plant survival and growth [46].

In addition to traditional seed priming, exogenous applications of osmoprotectants such as polyethylene glycol (PEG), proline, and glycine betaine have shown promising results in mitigating the effects of abiotic stress, including salinity. PEG is widely used to simulate drought and osmotic stress conditions and has been demonstrated to induce adaptive responses in plants by altering gene expression, enhancing antioxidant enzyme activities, and improving osmotic adjustment [47,48,49]. PEG-mediated priming can also influence hormonal signaling pathways and improve membrane stability, contributing to better seedling vigor and stress resilience [50,51,52]. Similarly, other exogenous compounds like salicylic acid, jasmonic acid, and melatonin have been reported to enhance salt tolerance by modulating antioxidant defenses and stress-responsive pathways [53,54,55]. These treatments offer complementary or synergistic effects when used alongside or in place of conventional seed priming techniques, broadening the scope of physiological and biochemical mechanisms that can be exploited for crop improvement under stress.

The level of reactive oxygen species (ROS) increases in plants facing salt stress and leads to cellular damage. Thus, to maintain cellular activity and cope with salt stress, plants have enzymatic and non-enzymatic antioxidants as a defense system. Plants have various enzymes to scavenge ROS [4]. Superoxide dismutase (SOD) converts superoxide radicals (O_2_^•–^) into hydrogen peroxide (H_2_O_2_) and oxygen (O_2_^•–^), then catalase (CAT) which is primarily found in peroxisomes breaks down H_2_O_2_ into water (H_2_O) and oxygen (O_2_^•–^) [56,57]. Ascorbate peroxidase (APX) uses ascorbate as an electron donor to reduce hydrogen peroxide to water. Monodehydroascorbate reductase (MDHAR) and dehydroascorbate reductase (DHAR) regenerate ascorbate from its oxidized forms, maintaining the ascorbate pool [58]. Therefore, this cycle will generate cell energy and mitigate the effect of ROS. Previous studies show that there is a link between salt stress and the plant antioxidant system [59]. Studies have also reported that an enhanced antioxidant system is directly linked to salt tolerance. Therefore, salt tolerance is directly related to the activity of antioxidants in plants, and especially in tomatoes has it been reported that SOD, CAT, and APX are essential antioxidants to regulate salt stress [60].

However, no study has been conducted about the effects of seed priming on enhancing the antioxidant activity of tomato plants under salt stress. Thus, this study hypothesized that seed priming with PEG6000 might enhance antioxidant capacity, thereby mitigating ROS damage under salt stress.

## 2. Results

### 2.1. Leaf Nutrient Analysis

**Figure 1:**
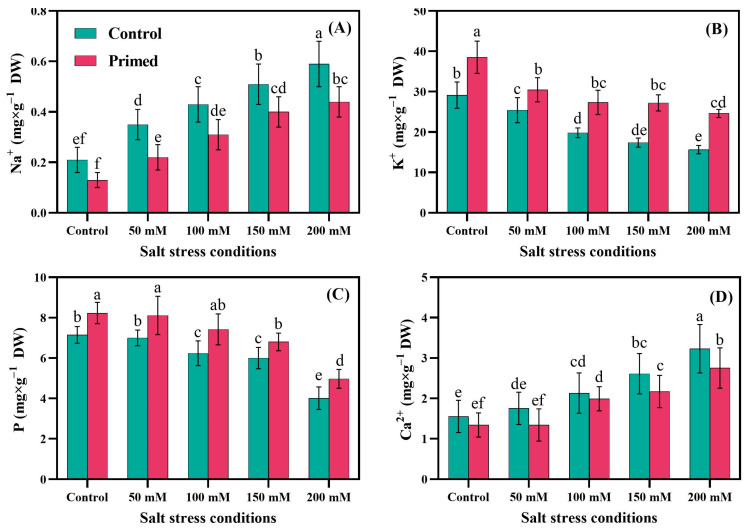
Concentrations of elemental ions in tomato leaf tissues under salt stress conditions. Panels show levels of sodium (**A**), potassium (**B**), phosphorus (**C**), and calcium (**D**). DW: dry weight. Different lowercase letters indicate statistically significant differences among treatments based on Tukey’s test at *p* < 0.05; same letters indicate no significant difference. Each treatment was replicated six times (*n* = 6).

Leaf element contents at the fruit-bearing stage of the tomato are shown in Figure 1. Salinity and priming had a highly significant impact on Na^+^, K^+^, P, and Ca^2+^ content. The interaction of salinity level and priming treatment had a significant effect on Na^+^ (*p* < 0.01), K^+^ (*p* < 0.01), P (*p* < 0.05), and Ca^2+^ (*p* <0.05). Compared to control (S_0_), seed priming treatment showed a significant increase in leaf sodium content by 66%, and this increment was rapid under 100 mM, 150 mM, and 200 mM NaCl. Seed priming treatment reduced leaf sodium content in all salinity treatments by 38%, 37%, 28%, 22%, and 25%, respectively. The variations in leaf Ca^2+^ content among treatments followed the same trend as that of sodium content, with the highest under 200 mM salinity and no priming and the lowest in 0 mM and 50 mM with primed seeds. Salinity reduced K^+^ significantly by 16%, 34%, 41%, and 48% under 50 mM, 100 mM, 150 mM, and 200 mM salinity, respectively. Seed priming treatment significantly increased K^+^ by 31%, 20%, 42%, 58%, and 60%, respectively. The highest leaf K^+^ was in 0 mM salinity with primed seeds and the lowest in 200 mM salinity with no seed priming. The same trend was observed in leaf P, with the highest P content in 0 mM salinity with primed seeds and the lowest in 200 mM salinity with no seed priming.

### 2.2. Fruit Quality Parameters

**Figure 2:**
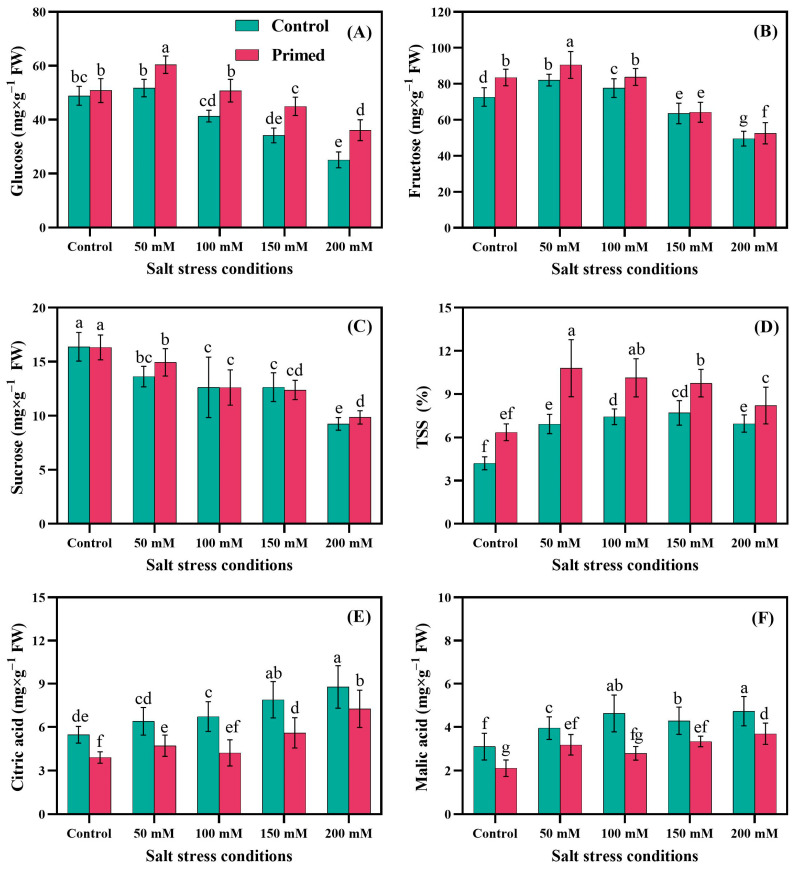
Enhancement of fruit quality parameters in tomato under salt stress conditions through seed priming. Panels show concentrations of glucose (**A**), fructose (**B**), sucrose (**C**), total soluble solids (**D**), citric acid (**E**), and malic acid (**F**). Different lowercase letters indicate statistically significant differences among treatments based on Tukey’s test at *p* < 0.05; same letters indicate no significant difference. Each treatment was replicated six times (*n* = 6).

As shown in Figure 2, salinity and seed priming have significant effects on the fruit sugar and acid content of the tomato fruit. With the increase in salinity level, the fruit sugar content increased in 50 mM NaCl (S_1_), especially in the seed priming treatment, then decreased in 100 mM, 150 mM, and 200 mM NaCl. The organic acid content increased with an increase in salinity level. The fruit glucose level was increased in 50 mM NaCl (S_1_) compared to control (S_0_), but decreased under higher salinity levels. Seed priming significantly increased the fruit glucose level by 4%, 18%, 21%, 29%, and 44% in 50 mM, 100 mM, 150 mM, and 200 mM NaCl, respectively. The fruit fructose content increased in 50 mM NaCl and 100 mM NaCl by 14% and 6%; however, it decreased in 150 mM and 200 mM NaCl by 12% and 32%. The seed priming treatment induced fruit fructose levels by 15% in S_0_P_1_, 10% in 50 mM NaCl with primed seeds (S_1_P_1_), 8% in 100 mM NaCl with primed seeds (S_2_P_1_), 2% in 150 mM NaCl with primed seeds (S_3_P_1_), and 6% in 200 mM with primed seeds (S_4_P_1_). The highest fruit fructose level was in 100 mM NaCl with primed seeds (S_1_P_1_), and the lowest was in 200 mM NaCl with no priming (S_4_P_0_). The same trend was observed in fruit sucrose levels, with the highest and lowest levels in 0 mM NaCl with no priming (S_0_P_0_) and 200 mM NaCl with no priming (S_4_P_0_)_,_ respectively. TSS was increased by an increment in the salinity level. Compared to no priming (P_0_), seed priming treatment (P_1_) increased total soluble solids (TSS) by 48%, 57%, 41%, 26%, and 29% in 0 mM, 50 mM, 100 mM, 150 mM, and 200 mM salinity, respectively. Both citric acid and malic acid increased with an increment in salinity level, while seed priming treatment significantly decreased them. The highest citric acid was in 200 mM NaCl with no priming (S_4_P_0_) while the lowest was in 100 mM NaCl with seed priming (S_2_P_1_), and in case of malic acid the highest concentration was observed in 200 mM NaCl with no priming (S_4_P_0_); however, 0 mM NaCl with seed priming (S_0_P_1_) had the lowest malic acid.

### 2.3. Cell Damage Indicators

Roots and leaves are often the first plant organs to exhibit stress symptoms. As shown in Table 1, in leaves, the abscisic acid level was significantly decreased with an increment in salinity level (*p* < 0.001). Compared to the control, salinity decreased leaf ABA levels by 18%, 24%, 45%, and 66% in 50 mM, 100 mM, 150 mM, and 200 mM NaCl, respectively. Seed priming treatment significantly increased ABA in leaves by 27%, 35%, 40%, 50%, and 81% in 0mM, 50 mM, 100 mM, 150 mM, and 200 mM NaCl, respectively. The highest leaf ABA level was in 0 mM NaCl with primed seeds (S_0_P_1_), the lowest was observed in 200 mM NaCl with no priming (S_4_P_0_). A similar trend was observed in root ABA levels when exposed to salinity, but increased in the seed priming treatment. The highest root ABA level was in 0 mM NaCl with primed seeds (S_0_P_1_), while the lowest was in 200 mM NaCl with no priming (S_4_P_0_). Salinity stress significantly induced (*p* < 0.001) leaf MDA and proline levels. However, seed priming treatment significantly decreased both MDA and proline levels (*p* < 0.001) in leaves. Compared to no priming, seed priming treatment decreased leaf MDA by 38%, 36%, 19%, 31%, and 25%, and leaf proline by 19%, 46%, 22%, 38%, and 35% in 0 mM, 50 mM, 100 mM, 150 mM, and 200 mM NaCl, respectively. Leaf electrolyte leakage (EL^L^) was also induced by an increment in the salinity level. Seed priming treatment significantly decreased by 9%, 11%, 11%, and 9% in 50 mM, 100 mM, 150 mM, and 200 mM NaCl, respectively. However, the effect of seed priming treatment was not significant on EL^L^ in 0 mM NaCl.

### 2.4. Antioxidant Enzymes and Redox Regulation

**Figure 3:**
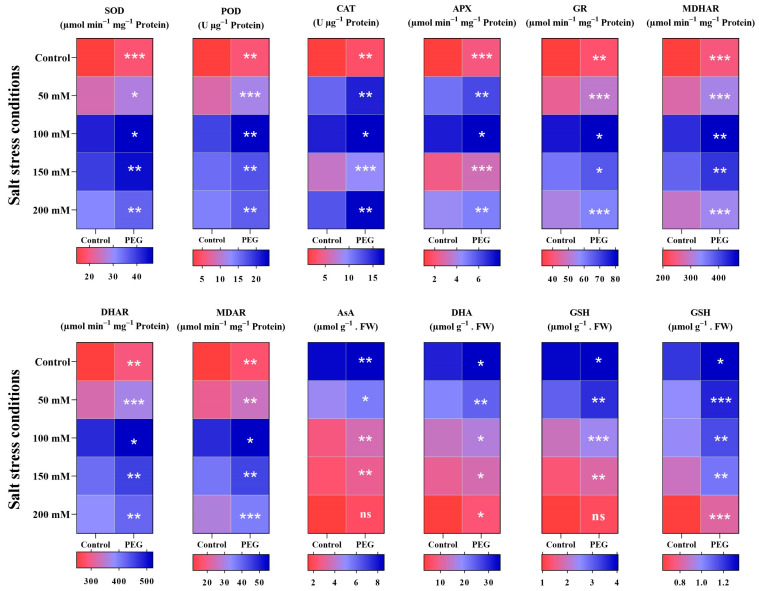
Antioxidant enzymes and redox regulation in tomato plants grown under salt conditions. The data are presented as the means of 6 replications (n = 6). Significance levels are denoted as *** for *p* < 0.001, ** for *p* < 0.01, and * for *p* < 0.05. SOD: Superoxidase Dismutase, POD: Peroxidase, CAT: Catalase, APX: Ascorbate Peroxidase, GR: Glutathione Reductase, MDHAR: Monodehydroascorbate Reductase, DHAR: Dehydroascorbate Reductase, MDAR: Mitochondrial Dehydroascorbate Reductase, AsA: Ascorbic Acid content, DHA: Dehydroascorbic Acid content, GSH: Glutathione content, GSSG: Oxidized Glutathione content.

Figure 3 shows the antioxidant enzymes and redox regulation in tomato plants grown under salt stress conditions. Compared to 0 mM NaCl (S_0_), salinity stress significantly induced the activities of antioxidant enzymes in tomato leaves. The activities of their antioxidant enzymes were higher in the seed priming treatment. Seed priming induced SOD activity by 28.19%, 16.34%, 10.07%, 12.31%, and 13.29% in 0 mM (S_0_), 50 mM NaCl (S_1_), 100 mM NaCl (S_2_), 150 mM NaCl (S_3_), and 200 mM NaCl (S_4_), respectively, with the highest and lowest level in S_2_P_1_ and S_0_P_0_. Seed priming induced POD activity by ×2.2, 44.89%, 28.43%, and 18.31% in 0 mM, 50 mM, 100 mM, and 200 mM NaCl, except in 150 mM NaCl, which was not significant. The highest POD activity was in 100 mM NaCl with primed seeds (S_2_P_1_), while the lowest was in 0 mM NaCl with no seed priming (S_0_P_0_). Seed priming treatment induced CAT activity in 0 mM, 50 mM, 150 mM, and 200 mM NaCl by ×2.19, 31.41%, 34.17%, and 37.03%, but its effectiveness in 100 mM NaCl was not significant. Treatments 200 mM NaCl with primed seeds (S_4_P_1_) and 0 mM NaCl with no priming (S_0_P_0_) had the lowest CAT activity.

### 2.5. Fruit Yield

**Figure 4:**
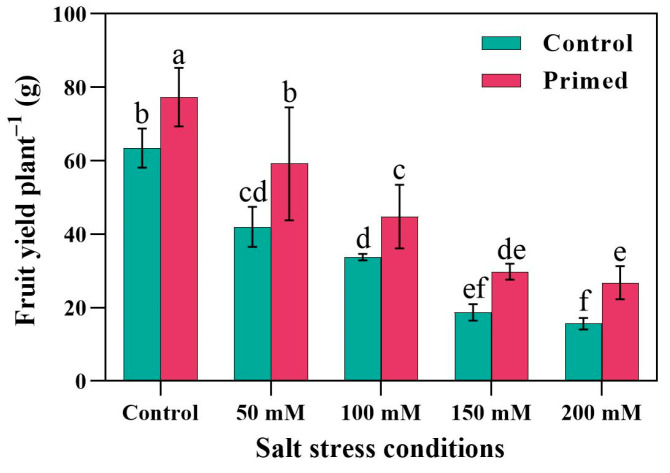
Impact of seed priming on fruit yield in tomato plants grown under salt conditions. The data are presented as means of 6 replications (n = 6). Different lowercase letters indicate statistically significant differences among treatments based on Tukey’s test at *p* < 0.05; same letters indicate no significant difference.

The analysis of fruit yield demonstrated that seed priming had a significant positive effect on fruit production across all salinity levels. Notably, the highest fruit yield was recorded in the control group (0 mM NaCl) where seeds were subjected to priming treatment. In contrast, the lowest fruit yield was observed under severe salt stress (200 mM NaCl) in the absence of seed priming. Across all tested salinity conditions, plants grown from primed seeds consistently outperformed those from non-primed seeds in terms of fruit yield, indicating the robustness of seed priming in mitigating the adverse effects of salt stress. These findings suggest that seed priming enhances the plant’s physiological capacity to cope with saline environments, thereby promoting better reproductive outcomes. The differences in fruit yield between primed and non-primed treatments were statistically significant at each salinity level (Figure 4), underscoring the potential of seed priming as an effective agronomic strategy to improve crop productivity under salt-affected conditions.

**Figure 5:**
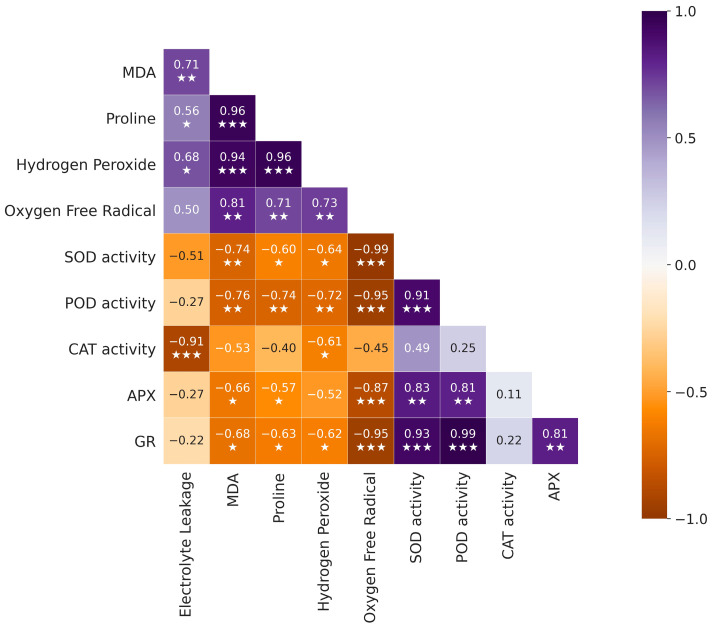
Correlation analysis of antioxidant enzymes and stress indicators. SOD: Superoxidase Dismutase, POD: Peroxidase, CAT: Catalase, APX: Ascorbate Peroxidase, GR: Glutathione Reductase. Significance correlations are denoted as *** for *p* < 0.001, ** for *p* < 0.01, and * for *p* < 0.05.

Based on the correlation analysis, there is a significant negative correlation between SOD and stress indicators, including MDA (*p* < 0.005), proline (*p* < 0.05), hydrogen peroxide (*p* < 0.05), and O_2_^•–^ (*p* < 0.001). Additionally, POD shows a significant negative correlation with MDA (*p* < 0.005), proline (*p* < 0.005), hydrogen peroxide (*p* < 0.005), and O_2_^•–^ (*p* < 0.001). Furthermore, CAT exhibits a significant negative correlation with electrolyte leakage (*p* < 0.001) and hydrogen peroxide (*p* < 0.05), while its negative correlation with MDA, proline, and O_2_^•–^ is not statistically significant. Moreover, APX and GR activity were revealed to have a negative relationship with MDA, proline, hydrogen peroxide, and oxygen free radicals (Figure 5).

APX was induced under salt stress compared to 0 mM NaCl (S_0_), and was higher in priming treatment by ×2, 18.18%, 11.06%, 35.55%, and 14.69% in 0 mM (S_0_), 50 mM (S_1_), 100 mM (S_2_), 150 mM (S_3_), and 200 mM NaCl (S_4_), respectively. The highest and lowest APX activity was in 100 mM NaCl with primed seeds (S_2_P_1_) and 0 mM NaCl with no priming (S_0_P_0_). GR, MDHAR, DHAR, and MDAR activities were enhanced by seed priming treatment except in 100 mM NaCl (S_2_) and 150 mM NaCl (S_3_); the seed priming effect was not significant. Seed priming treatment induced GR by 16.45%, 16.13%, 6.22%, 7.76%, and 11.48%, MDHAR increased by 21.08%, 16.71%, 11.09%, 11.29%, and 10.52%, DHAR increased by 16.70%, 12.95%, 9.16%, 10.03%, and 10.68%, MDAR increased by 43.74%, 25.04%, 14.69%, 20%, and 23.21% in 0 mM (S_0_), 50 mM (S_1_), 100 mM (S_2_), 150 mM (S_3_), and 200 mM NaCl (S_4_), respectively. The highest and lowest levels of GR were in 150 mM NaCl with primed seeds (S_3_P_1_) and 0 mM NaCl with no priming (S_0_P_0_), for MDHAR were in 100 mM NaCl with primed seeds (S_2_P_1_) and S_0_P_0_, for DHAR were in 100 mM NaCl with primed seeds (S_2_P_1_) and 0 mM with no priming (S_0_P_0_), and for MDAR were in 100 mM NaCl with primed seeds (S_2_P_1_) and 0 mM NaCl with no priming (S_0_P_0_). Asa, DHA, GSH, and GSSH decreased under salt stress but were significantly induced by seed priming. The highest and lowest AsA, DHA, GSH, and GSSH were in 0 mM NaCl with primed seeds (S_0_P_1_) and 200 mM NaCl with no priming (S_4_P_0_), respectively.

Tomatoes respond to salt stress through various physiological and biochemical mechanisms. Among them, the roles of Diamine Oxidase (DAO), Polyamine Oxidase (PAO), and Arginine Decarboxylase (ADC) are particularly significant due to their involvement in polyamine metabolism and oxidative stress response. DAO significantly decreased under salt stress, while seed priming treatment significantly induced it. The highest and lowest DAO were in 0 mM NaCl with primed seeds (S_0_P_1_; 92.01) and 200 mM NaCl with no priming (S_4_P_0_; 26.18), respectively. The same trend was observed in PAO and ADC. The highest PAO and ADC were recorded at 150.57 and 139.14 in 0 mM NaCl with primed seeds (S_0_P_1_), while the lowest were at 55.14 and 44.25 in 200 mM NaCl with no priming (S_4_P_0_) (Table 2).

Table 3 reveals that salt stress affects some physiological and biomarkers, too. The variance analysis shows that tomato plants’ root activity is reduced under salt stress conditions. Plants under salt stress produced shorter and less active roots compared to the control. Salt stress decreased root activity 10.41%, 18.57%, 41.75%, and 55.89% in 50 mM (S_1_), 100 mM (S_2_), 150 mM (S_3_), and 200 mM NaCl (S_4_) compared to 0 mM NaCl (S_0_). The highest and lowest root activity was observed in 0 mM NaCl with primed seeds (S_0_P_1_; 37.78) and 200 mM NaCl with no priming (S_4_P_0_; 11.69). The membrane solubility index is one of the indicators that display cell tolerance against stress. Our findings indicate that MSI was drastically decreased by salt stress, but seed priming significantly induced it. The highest and lowest MSIs were in 0 mM NaCl with primed seeds (S_0_P_1_) and 200 mM NaCl with no priming (S_4_P_0_). The protein, which plays a vital role in the detoxification of sodium ions, was decreased under salt stress in both tomato leaves and roots. However, seed priming treatment significantly induced protein in leaves and roots. The highest and lowest proteins in leaves were 17.65 and 1.21, and in roots were 2.79 and 0.15, which were observed in 0 mM NaCl with primed seeds (S_0_P_1_) and 200 mM NaCl with no priming (S_4_P_0_). ROS plays a crucial role in plant responses to salt stress by acting as signaling molecules and regulators of stress-related pathways. However, their excessive accumulation can lead to oxidative damage. In this experiment, we observed that stress significantly induced ROS (H_2_O_2_ and O_2_^•–^), but seed priming treatment significantly decreased it. The highest and lowest H_2_O_2_ were 34.61 and 5.31, and the highest and lowest O_2_^•–^ were 20.61 and 0.99, which were recorded in 200 mM NaCl with no priming (S_4_P_0_) and 0 mM NaCl with no priming (S_0_P_0_) for both H_2_O_2_ and O_2_^•–^. WP, OP, and TP were significantly induced under salt stress conditions, while seed priming treatment decreased them. The highest and lowest WP, OP, and TP were in 200 mM NaCl with no priming (S_4_P_0_) and 0 mM NaCl with primed seeds (S_0_P_1_). WP ranged from 1.08 to 5.86, OP ranged from –0.26 to –1.48, and TP ranged from 0.42 to 1.68. Salt stress significantly decreased RWC in tomato plant tissues, while plants under seed priming treatment were fleshy and had significantly higher RWC compared to the control. Salt stress decreased to RWC 14.59%, 24.07%, 33.38%, and 50.14% in 50 mM (S_1_), 100 mM (S_2_), 150 mM (S_3_), and 200 mM NaCl (S_4_), respectively.

## 3. Discussion

### 3.1. Nutrient Composition in Leaf

Salt stress is a significant barrier to plant growth and development [61,62]. It inhibits the shoot and root growth through different mechanisms [34]. Seed priming proved to be useful for promoting various growth, physiological, and yield parameters under salt stress conditions [6,9]. In this study, we found that seed priming enhances nutrient composition in tomato leaves (Figure 1). Elemental nutrient analysis of tomato plant leaves showed that Na^+^ and Ca^+^ were induced by salt stress, but P and K^+^ were decreased under salt stress conditions, which similar finding reported by Guo et al. [63] and Parvin et al. [64]. We also found that seed priming with PEG6000 enhanced P and K^+^ while a decrement in Na^+^ and Ca^2+^ concentration was observed in tomato leaves compared to non-primed plants. No previous study investigated the impact of seed priming with PEG6000 on P, K^+^, Na^+^, and Ca^2+^. Under NaCl salt stress, Na^+^ replaces Ca^2+^ in the cell wall, which causes damage and electrons leak as K^+^. Furthermore, phosphorus is also important because it is a core component of ATP (adenosine triphosphate), which is the energy currency of the plant cell.

### 3.2. Fruit Quality Parameters

The effect of seed priming on fruit quality parameters such as sugars (glucose, fructose, sucrose, and TSS), and organic acids (malic acid and citric acid) under salt stress conditions (Figure 2) revealed that salt stress in small amounts improves sugars, but high salt stress drastically decreases it; however, seed priming with polyethylene glycol induces sugars but decreases acid content compared to control. It was previously reported that seed priming with PEG6000 can increase the fruit quality in tomato plants under salt stress [6].

### 3.3. Cell Damage Indicators

Osmotic regulation can play a vital role in plant defense against salt stress. In this process, plants produce some substances to increase their tolerance to stressful conditions [65]. Cell damage indicators such as ABA in leaves and roots, MDA, and proline in tomato leaves were evaluated to check injury levels due to salt stress and recovery as the effect of seed priming treatment. ABA showed a negative response toward salt stress as its concentration decreased in leaves and root tissues with an increase in salt stress level. ABA concentration in roots was in parallel with root activity (Table 1). The results revealed a decrease in ABA concentration in roots, followed by a decrease in root activity. ABA concentration in both leaves and roots was induced under the seed priming treatment compared to the control. The highest ABA concentration was under ambient conditions and in plants derived from primed seeds, where the lowest concentration was determined in S_4_. Furthermore, tomato roots had a higher concentration of ABA compared to their shoots. Raziq et al. [66] reported ABA levels in the roots and leaves of seedlings that were stressed for 12 days were significantly higher than those of normal seedlings, and ABA ranged from ~47 to 73 in leaves and ~40 to 80 in roots. We observed the opposite trend. We found that salt stress decreased the ABA concentration in leaves and roots, while seed priming significantly induced it. In the current study, ABA amounts varied from 21.36 to 79.77 in leaves and 20.59 to 84.16 in roots. This result shows that seed priming promotes ABA in leaves and roots. MDA, proline, and EL were increased by an increment in salt stress level. Increased levels of MDA, proline, and EL indicate cell membrane injury. This injury might be oxidative stress and higher levels of H_2_O_2_ and O_2_^•–^ ROS (Table 1) which are caused by salt stress. Furthermore, higher EL indicates the cell membrane is damaged, and K^+^ ions have leaked, and therefore the tissues might be lacking in K^+^ concentration (Table 1). Adhikari et al. [67] evaluated the effect of seed priming on lettuce yield under salt stress and reported that seed hydropriming significantly decreased MDA in 0 mM and 100 mM NaCl salt stress, while the lowest EL and proline were observed in hydropriming and non-priming treatment, respectively. The current study confirms Adhikari et al. [67]. Their results indicated that seed priming decreases MDA and EL, while, in the current study, only PEG6000 is used as a seed priming reagent. Moreover, we observed that seed priming significantly decreases proline content compared to non-primed plants which is contrary to Adhikari et al. [67].

### 3.4. Antioxidant Enzyme Activity

Plants facing salt stress can develop various mechanisms to mitigate the negative effects. Enzymatic activity is the primary reaction of plants to stressful environments [68,69]. In our current study, we evaluated the positive effects of seed priming on enzymatic activity in tomatoes under salt stress. Enzymes such as SOD, POD, CAT, APX, GR, MDHAR, DHAR, and MDAR were induced under salt stress, and it was observed that salt stress from 50 mM to 150 mM NaCl promoted enzymatic activity; however, 200 mM NaCl reduced it (Figure 3), while enzymatic activity was higher under seed priming treatment compared to non-primed plants. Aydin [70] also reported that enzymatic activity significantly increased under salt stress compared to the control, and 88.87%, 204.39%, 83.87%, 157.29%, and 88.23% increases were reported in SOD, POD, CAT, APX, and GR, respectively. The current study differs in terms of salt type and stress level because Aydin [70] used one salt level, while, in the current study, four salt levels were used, and our results indicate that high salt stress significantly reduces enzymatic activity. Furthermore, the current study suggests that seed priming is useful for inducing enzymatic activity, which indicates the difference from the Aydin [70] study. Salt stress was reported to increase SOD, POD, CAT, and APX [71], which the current study confirms; however, Pérez-Labrada et al. [71] used foliar copper nanoparticles to promote salt stress while in the current study seed priming was used to induce tolerance. The current study showed that AsA, DHA, GSH, and GSSG had an opposite trend compared to enzymatic activity (Figure 3). It was observed that salt stress decreased AsA, DHA, GSG, and GSSG compared to the control, but seed priming significantly induced AsA, DHA, GSG, and GSSG, which no previous study reported.

Polyamine enzymes (DAO, PAO, and ADC) ensure the tight regulation of polyamine metabolism, which is crucial for maintaining cellular homeostasis and responding to physiological and environmental changes [72]. These enzymes are essential for the adaptation of plants to stress conditions such as salinity [73]. It has been found that polyamine enzyme levels change under salt stress and stress duration [74,75,76]. DAO, PAO, and ADC levels decrease in tomato roots under salt stress, showing a difference of –71.54% (DAO), –59.65% (PAO), and –64,65% (ADC) in 200 mM NaCl salt stress compared to the control (Table 2). DAO, PAO, and ADC were promoted in tomato roots under seed priming treatment. DAO activity was elevated by 15.09%, 16,23%, 25.14%, 30.90%, and 53.71%, similarly PAO activity increased 10.17%, 13.95%, 13,61%, 17.76%, and 26.44%, and ADC activity increased 11.13%, 12.65%, 15.81%, 19.82%, and 36.11% in seed priming under 0 mM, 50 mM, 100 mM, 150 mM, and 200 mM NaCl salt stress, respectively.

In high salt stress, the cell membrane stability index is restricted due to the accumulation of Na^+^ and the low ratio of K^+^/Na^+^ [18]. Na^+^ accumulation in leaves (Table 1) caused MSI (membrane solubility index) to decrease (Table 3). This might be due to low K^+^ concentration, cell membrane injury, and restricted functionality of channel proteins, which previous studies confirm [77]. We found that the protein content of the tomato leaf and root significantly decreased under salt stress conditions, but it was induced by seed priming treatment. Protein decrement under salt stress might be due to the degradation of Rubisco, and seed priming might be effective in avoiding Rubisco degradation. Khalifa [78] reported that although the protein accumulation under salt stress differs from plant to plant it significantly decreased in tomato plants. The other reason for restricted MSI and protein accumulation might be the induced ROS (H_2_O_2_, and O_2_^•–^) under salt stress conditions. We found that H_2_O_2_ and O_2_^•–^ were significantly induced under salt stress, and, alongside that, protein accumulation decreased both in tomato leaves and roots. We also observed that salt stress disturbed the plant–water relationship. The results revealed that relative water content drastically decreased in plants facing high salt stress (Table 3). Furthermore, water potential became more negative due to salt stress. This was along with high osmotic potential and turgor potential. Zhao et al. [79] also reported that salt stress regulated osmotic pressure by lowering the osmotic potential. Similarly, Perez-Lopez et al. [13] reported that osmotic potential was reduced under salt stress. The results from this study show that seed priming can be a beneficial and easy practice for farmers to diminish the negative effects of salt stress on their crop yield (Figure 4). Moreover, future research on DNA and genome analysis is required to check the impacts of seed priming.

## 4. Materials and Methods

### 4.1. Plant Materials and Treatments

This experiment took place in a controlled greenhouse at Tokyo University’s Setagaya Campus. Seeds of the tomato cultivar *Solanum lycopersicum* cv. Micro-Tom (wild type) was selected for this study. The study followed a factorial completely randomized design (CRD) with five salinity treatments and two priming treatments, each combination replicated six times. With 300 plants in total, the study was both comprehensive and meticulously designed. Polyethylene glycol (PEG6000) (Cica-Reagent, Tokyo, Japan) was used to prime the seeds by soaking them in a solution for 48 h. After soaking, the seeds were washed three times with clean water, dried on a table for three hours, and then weighed to estimate their initial weight. PEG6000 was applied at a concentration of P_0_: 0 MPa and P_1_: −1.2 MPa, determined using the below formula [26,80]:

OP = (−1.18 × 10^−2^) × C − (1.18 × 10^−4^) × C + (2.67 × 10^−4^) × C × T + (8.39×10^−7^) × C^2^T (1)

In the above equation, OP is osmotic pressure, T is temperature, and C is PEG concentration. For salinity stress, sodium chloride was used at concentrations of S_0_: 0 mM, S_1_: 50 mM, S_2_: 100 mM, S_3_: 150 mM, and S_4_: 200 mM. Seeds were initially sown in trays and transplanted into rock wool cubes (soilless culture) after a month. Salinity treatments began when seedlings had 3–4 leaves and continued until fruit harvest. The irrigation water, adjusted to a pH of 5.8–6 with sodium hydroxide and hydrochloric acid, included sodium chloride for desired salinity levels. Fresh irrigation water was prepared for each cycle to prevent salt accumulation, ensuring consistent treatment for all plants.

The choice of polyethylene glycol 6000 (PEG6000) in this study was based on its well-documented role as an effective osmotic agent used to simulate water-deficient and salinity-like stress conditions in plants. PEG6000 is a high-molecular-weight, non-penetrating polymer that induces osmotic stress externally without being absorbed by plant tissues, making it an ideal agent to mimic drought and salinity stresses in a controlled manner. Its inert nature prevents direct toxic effects on seeds or seedlings, thereby allowing researchers to investigate the physiological and biochemical responses to osmotic stress alone.

The concentrations of PEG6000 used in this study were carefully selected based on the previous literature demonstrating optimal priming effects within a moderate osmotic potential range. Concentrations such as 10%, 15%, and 20% PEG6000 correspond to specific osmotic potentials that have been shown to enhance germination, early seedling vigor, and antioxidant enzyme activities without imposing irreversible damage. These concentrations were chosen to capture a gradient of stress responses and identify the most effective dose for priming tomato seeds.

This range allowed us to evaluate both the threshold and potential benefits of PEG priming on enhancing salinity tolerance through antioxidant defense mechanisms. Notably, the 15% concentration emerged as particularly effective, as it balanced between eliciting a strong priming response and avoiding excessive stress that could impair seedling establishment.

During the experiment, tomato plants were grown under controlled greenhouse conditions to ensure optimal growth and minimize environmental variability. The greenhouse was maintained at 25 ± 2 °C during the day and 18 ± 2 °C at night, which aligns with the ideal temperature range for tomato development. Relative humidity was kept between 60% and 70%, ensuring proper transpiration and minimizing stress from water loss.

The photoperiod was adjusted to simulate long-day conditions, with 16 h of light and 8 h of darkness per day. Supplemental lighting was provided using high-pressure sodium (HPS) lamps, delivering a light intensity of approximately 400–600 µmol·m⁻^2^·s⁻^1^ at canopy level. These conditions are optimal for photosynthesis and fruit development in tomato plants.

To support vigorous plant growth, a balanced nutrient solution was applied throughout the experiment. Plants received nutrients based on a standard tomato fertilization regime using a modified Hoagland’s solution. The nutrient composition included Nitrogen (N): 14 mM (as NO_3_⁻ and NH_4_^+^), Phosphorus (P): 1.0 mM (as H_2_PO_4_⁻), Potassium (K): 6.0 mM (as K^+^), Calcium (Ca): 4.0 mM (as Ca^2+^), Magnesium (Mg): 2.0 mM (as Mg^2+^), Sulfur (S): 2.0 mM (as SO_4_^2^⁻), Micronutrients: Iron (Fe-EDTA), manganese (Mn), zinc (Zn), copper (Cu), boron (B), and molybdenum (Mo) in appropriate concentrations.

Nutrient solution was applied twice per week through a drip irrigation system, ensuring consistent nutrient availability. Electrical conductivity (EC) and pH of the nutrient solution were monitored and maintained at 2.0–2.5 dS/m and 5.8–6.2, respectively.

These environmental and nutritional conditions were maintained uniformly across all treatment groups, including both control and salt-stressed plants, to isolate the effects of salinity and seed priming on tomato growth and yield.

### 4.2. Determination of Antioxidant Components

In our study, we analyzed leaf nutrient elements, encompassing sodium (Na), potassium (K), phosphorus (P), and calcium (Ca), employing the methodology outlined by [6,9]. Six fruits per treatment were used for the quantification of fruit sugars, including glucose, sucrose, and fructose, as well as organic acids such as malic and citric acids. The quantification was carried out using HPLC as per [81]. The protein content of leaves and roots was extracted from six samples each using the fresh tissues. The quantification followed the procedure by Han et al. [82].

For the assay of antioxidant enzymes, 0.2 g of fresh leaf tissues was homogenized in phosphate buffer (50 mM, pH 7.8) in a pre-chilled mortar, followed by centrifugation (12,000× *g* at 4 °C) for 20 min. The samples were stored at −80 °C for further analysis. SOD activity was checked by using nitro blue tetrazolium (NBT) [83]. Half (50%) inhibition of photoreduction of NBT by enzyme activity was considered as one unit of SOD activity. POD activity was measured by the oxidation of guaiacol in a reaction mixture containing phosphate buffer (0.2 M, pH 6.0), guaiacol (50 mM), and hydrogen peroxide (2%), and absorbance was checked at 470 nm. CAT was measured according to the previously described protocols by the reduction in H_2_O_2_ as the decrease in absorbance at 240 nm [84,85]. APX activity was initiated by combining a fraction of the enzyme extract with a mixture containing phosphate buffer (50 mM, pH 7.0), 0.1 mM EDTA, 0.1 mM H_2_O_2_, and 0.5 mM ascorbate [86]. The change in absorbance was measured at 290 nm. GR activity was assessed using a specific kit from Solarbio Life Science, Beijing, China, following the manufacturer’s instructions. MDHAR (monodehydroascorbate reductase) and DHAR (dehydroascorbate reductase) were determined based on previously established protocols, with absorbance readings at 340 nm and 265 nm, respectively. For non-enzymatic antioxidants, such as ascorbate (AsA-DHA) and glutathione (GSH-GSSG), samples were prepared by homogenizing 0.2 g of leaf tissue in 6% pre-chilled HClO_4_. The supernatant obtained after centrifugation (12,000× *g* for 15 min at 4 °C) was stored at −80 °C for subsequent analysis. The contents of AsA-DHA and GSH-GSSG were then determined based on the method previously described [87,88,89]. To measure PAO activity, fresh leaf tissue was homogenized in 100 mM phosphate buffer (pH 6.5), and the homogenate was centrifuged at 10,000× g for 20 min at 4 °C. The reaction mixture consisted of 100 mM phosphate buffer (pH 6.5), 200 µL of a 4-aminoantipyrine/N, N-dimethylaniline solution, 100 µL of horseradish peroxidase, and 200 µL of enzyme extract. The reaction was initiated by adding 20 mM of both spermidine and spermine, and the change in optical density was measured at 254 nm [87].

### 4.3. Quantification of ABA in Shoot and Root Cells

ABA levels were evaluated in tomato shoots and roots. Quantification of ABA was conducted using a highly sensitive indirect ELISA method [90], enabling the precise measurement of abscisic acid levels. To enhance the accuracy of the assay, ABA-BSA conjugates were meticulously prepared according to the protocol outlined in Weiler [91] and Norman et al. [92]. ABA was measured using fresh tissues, and the final ABA levels are expressed in ng/g FW.

### 4.4. Determination of Reactive Oxygen Species (ROS)

Hydrogen peroxide (H_2_O_2_) was measured from 50 mg of fresh leaf material extracted with a 0.1% (*w*/*v*) trichloroacetic acid (TCA) solution, followed by centrifuging the extract. The supernatant was mixed (1:1) with potassium phosphate buffer (10 mM, pH 7.0) and (1:2) KI (1 M). The absorbance was measured at 390 nm [93]. Concentrations were calculated against an H_2_O_2_ standard calibration curve and expressed as µmol g^−1^ DW. O_2_^•–^ was measured by mixing the supernatant of the processed leaf sample with phosphate buffer (50 mM: pH 7.8) and hydroxylamine hydrochloride (10 mM). The absorbance was checked at 530 after incubating the incubation of reaction mixture incubation for about half an half-hour at room temperature. For O_2_^•–^, the leaf sample was processed in TCA (0.1%) and centrifuged (12,000× *g*; 15 min; 4 °C). The obtained supernatant was mixed with KI (1 M) and phosphate buffer (0.1 M; pH 7.8), and absorbance was noted at 390 nm after a dark incubation for one hour [94].

### 4.5. Determination of Stress Indicators

Malondialdehyde (MDA) and proline were measured from six random leaves of different plants in each treatment. The procedure followed the protocol as previously described [93,95,96]. MDA contents were expressed as nmol g^−1^ DW, and proline content as µmol g^−1^ FW. Electrolyte leakage was assessed by determining the extent of leaf damage by measuring the number of electrons that were leaking from the leaves [6,97].

### 4.6. Determination of Water Potential in Plant Tissues

The plant–water relations were calculated based on the procedure explained in Raziq et al. [66] and Morgan [98] using the below equation:

RWC = (Fresh weight − Dry weight)/(Saturated weight − Dry weight) × 100(2)

### 4.7. Statistical Analysis

The data were analyzed using ANOVA with the statistical software R 3.6.2. To distinguish treatment means at 0.1%, 1%, and 5% significance levels, Tukey’s test was applied. For data visualization of the examined parameters, Python v.3.13 was employed.

## 5. Conclusions

The influence of seed priming on the antioxidant activity of tomato plants under salt stress was investigated. The results confirmed that seed priming with PEG6000 effectively enhances antioxidant activity, mitigating the impact of reactive oxygen species (ROS) under salt stress. Seed priming positively influenced fruit quality, protein accumulation in leaves and roots, and the activity of antioxidant enzymes. The consistent increase in antioxidant capacity across fruits, leaves, and roots demonstrated that seed priming significantly bolstered the plants’ ability to counteract oxidative stress. These findings support the hypothesis that seed priming enhances antioxidant activity, providing a mechanism to mitigate ROS and improve the salt tolerance of tomato plants. Seed priming with PEG6000 can be used as a practical strategy to reduce the harmful effects of salt stress, improving both yield and fruit quality. Furthermore, selecting the appropriate priming agent and concentration is crucial for maximizing the benefits of this technique in agricultural practices.

## Figures and Tables

**Table 1:** The influence of seed priming on the levels of leaf and root ABA, leaf MDA, proline, and electrolyte leakage in tomato plants under salt stress conditions.

Treatment	ABA^L^ (µg g^−1^ Protein)	ABA^R^ (µg g^−1^ Protein)	MDA^L^ (nmol g^−1^ DW)	Proline^L^ (µmol g^−1^ FW)	EL^L^ (%)
S_0_P_0_	62.34 ± 4.33 d	68.39 ± 3.76 cd	3.69 ± 0.46 e	2.89 ± 0.16 ef	29.95 ± 3.16 f
S_0_P_1_	79.77 ± 7.71 a	84.16 ± 4.92 a	2.27 ± 0.14 f	2.34 ± 0.11 f	28.89 ± 2.75 f
S_1_P_0_	51.73 ± 2.09 e	59.93 ± 2.18 e	6.77 ± 0.64 d	4.58 ± 0.29 de	38.77 ± 4.32 c
S_1_P_1_	69.16 ± 6.78 b	75.70 ± 3.75 b	4.35 ± 0.37 e	2.45 ± 0.13 f	35.12 ± 4.72 de
S_2_P_0_	45.95 ± 3.15 f	49.74 ± 2.80 f	9.15 ± 1.21 c	5.13 ± 0.41 bc	39.98 ± 5.12 c
S_2_P_1_	63.39 ± 4.22 cd	66.51 ± 2.64 d	7.18 ± 0.95 d	4.01 ± 0.12 f	35.68 ± 4.48 de
S_3_P_0_	34.26 ± 2.11 h	35.86 ± 1.66 g	13.66 ± 1.76 b	6.12 ± 0.25 b	43.15 ± 7.43 b
S_3_P_1_	51.67 ± 3.96 e	52.30 ± 4.87 f	9.42 ± 1.32 c	3.78 ± 0.13 cd	38.25 ± 4.86 cd
S_4_P_0_	21.36 ± 1.60 i	20.59 ± 1.33 h	21.05 ± 3.14 a	8.41 ± 0.72 a	47.76 ± 6.74 a
S_4_P_1_	38.8 ± 3.75 gh	36.70 ± 3.90 g	15.77 ± 2.28 b	5.46 ± 0.32 de	43.49 ± 5.23 b
*p* value					
S	***	***	***	***	***
P	***	***	***	***	*
S × P	***	***	***	***	**

The data are presented as mean and S.E., and each treatment was replicated 6 times (n = 6). S_0_: 0 mM NaCl, S_1_: 50 mM NaCl, S_2_: 100 mM NaCl, S_3_: 150 mM NaCl, S_4_: 200 mM NaCl, P_0_: 0 MPa, P_1_: 1.5 MPa PEG6000, ABA^L^: Abscisic Acid content in leaf, ABA^R^: Abscisic Acid content in root, MDA^L^: Malondialdehyde content in leaf, Proline^L^: Proline content in leaf, EL^L^: Electrolyte Leakage in leaf tissues. Different lowercase letters indicate statistically significant differences among treatments based on Tukey’s test at *p* < 0.05; same letters indicate no significant difference. Each treatment was replicated six times (*n* = 6). Significance levels in the interactions are denoted as *** for *p* < 0.001, ** for *p* < 0.01, and * for *p* < 0.05.

**Table 2:** Effect of seed priming on polyamine metabolism and growth regulation of tomato plants under salt stress conditions.

Treatment	DAO (U mg^−1^ Protein)	PAO (U mg^−1^ Protein)	ADC (U mg^−1^ Protein)
S_0_P_0_	92.01 ± 5.45 b	136.66 ± 6.34 b	125.21 ± 7.61 b
S_0_P_1_	105.90 ± 6.86 a	150.57 ± 5.22 a	139.14 ± 4.98 a
S_1_P_0_	73.54 ± 4.32 de	116.33 ± 4.76	105.78 ± 2.34 de
S_1_P_1_	85.48 ± 3.21 c	130.23 ± 5.12 c	119.16 ± 4.62 c
S_2_P_0_	55.33 ± 3.18 g	96.73 ± 3.25 of	89.67 ± 2.49 f
S_2_P_1_	69.24 ± 2.55 e	109.90 ± 4.11 d	103.85 ± 3.85 e
S_3_P_0_	45.01 ± 2.63 h	77.44 ± 2.65 g	70.02 ± 1.76 h
S_3_P_1_	58.92 ± 2.11 fg	91.20 ± 3.78 f	83.90 ± 1.99 g
S_4_P_0_	26.18 ± 1.05 j	55.14 ± 1.23 i	44.25 ± 1.15 j
S_4_P_1_	40.24 ± 1.48 i	69.72 ± 1.72 h	60.23 ± 1.77 i
*p* value			
S	***	**	**
P	**	*	*
S × P	**	*	*

The data are presented as mean and S.E., and each treatment was replicated 6 times (n = 6). Different lowercase letters indicate statistically significant differences among treatments based on Tukey’s test at *p* < 0.05; same letters indicate no significant difference. Significance levels in the interactions are denoted as *** for *p* < 0.001, ** for *p* < 0.01, and * for *p* < 0.05. S_0_: 0 mM NaCl, S_1_: 50 mM NaCl, S_2_: 100 mM NaCl, S_3_: 150 mM NaCl, S_4_: 200 mM NaCl, P_0_: 0 MPa, P_1_: 1.5 MPa PEG6000, DAO: Diamine Oxidase, PAO: Polyamine Oxidase, ADC: Arginine Decarboxylase.

**Table 3:** Effect of seed priming on some physiological parameters and biomarkers in tomato plants grown under salt stress conditions.

Treatment	RA (µg g^−1^·FW)	MSI (%)	LP (mg g^−1^·FW)	RP (mg g^−1^·FW)	H_2_O_2_ (µmol g^−1^·FW)	O_2_^•–^ (µmol g^−1^·FW)	WP (Mpa)	OP (Mpa)	TP (Mpa)	RWC (%)
S_0_P_0_	26.23 c	69.20 c	11.07 c	1.22 cd	5.31 h	3.17 e	−1.93 a	−0.52 ab	0.81 de	77.27 ab
S_0_P_1_	37.78 a	81.17 a	17.65 a	2.79 a	3.25 i	0.99 f	−1.08 a	−0.26 a	0.42 e	84.66 a
S_1_P_0_	23.50 d	63.80 d	7.15 e	0.83 e	10.82 e	7.22 d	−3.67 bc	−0.87 bcd	1.22 bc	65.99 cd
S_1_P_1_	36.16 a	76.57 b	13.41 b	2.52 b	7.12 g	3.81 e	−1.61 a	−0.71 ab	0.81 cde	74.41 bc
S_2_P_0_	21.36 e	44.80 f	3.22 h	0.54 f	16.33 d	14.85 c	−3.46 bc	−1.07 cde	1.27 ab	58.67 def
S_2_P_1_	33.74 b	56.60 e	9.02 d	2.18 bc	9.04 f	9.48 e	−2.22 ab	−0.75 bc	0.84 cde	68.19 bcd
S_3_P_0_	15.28 f	26.83 i	2.29 i	0.31 g	25.47 b	17.33 b	−4.84 de	−1.35 of	1.44 ab	51.48 ef
S_3_P_1_	20.15 e	35.54 g	4.98 g	1.13 d	16.33 d	12.89 d	−4.21 cd	−1.02 cde	1.10 bcd	60.72 de
S_4_P_0_	11.69 g	15.07 j	1.21 j	0.15 h	34.61 a	20.61 a	−5.86 e	−1.48 ef	1.68 a	38.53 g
S_4_P_1_	15.53 f	28.12 h	6.74 f	1.37 c	22.63 c	13.89 cd	−3.62 cd	−1.22 f	1.38 ab	50.67 f
*p* value										
S	***	***	***	***	***	***	***	***	***	***
P	***	***	***	***	***	***	***	***	**	**
S × P	***	***	**	**	***	*	*	*	*	*

The data are presented as the mean of 6 replications (n = 6). Different lowercase letters indicate statistically significant differences among treatments based on Tukey’s test at *p* < 0.05; same letters indicate no significant difference. Significance levels in the interactions are denoted as *** for *p* < 0.001, ** for *p* < 0.01, and * for *p* < 0.05. S_0_: 0 mM NaCl, S_1_: 50 mM NaCl, S_2_: 100 mM NaCl, S_3_: 150 mM NaCl, S_4_: 200 mM NaCl, P_0_: 0 MPa, P_1_: 1.5 MPa PEG6000, RA: Root Activity, MSI: Membrane Solubility Index, PL: Protein content in Leaf, PR: Protein content in Root, H_2_O_2_: Hydrogen peroxide, O_2_^•–^: Oxygen free radical, WP: Water Potential, OP: Osmotic Potential, TP: Turgor Potential, RWC: Relative Water Content.

## Data Availability

Data will be available upon request from the corresponding author.

## References

[1] González-García Y., López-Vargas E.R., Pérez-Álvarez M., Cadenas-Pliego G., Benavides-Mendoza A., Valdés-Reyna J., Pérez-Labrada F., Juárez-Maldonado A. (2022). Seed Priming with Carbon Nanomaterials Improves the Bioactive Compounds of Tomato Plants under Saline Stress. Plants.

[2] Tommonaro G., Abbamondi G.R., Nicolaus B., Poli A., D’Angelo C., Iodice C., De Prisco R. (2021). Productivity and Nutritional Trait Improvements of Different Tomatoes Cultivated with Effective Microorganisms Technology. Agric..

[3] Malik A., Mor V.S., Tokas J., Punia H., Malik S., Malik K., Sangwan S., Tomar S., Singh P., Singh N. (2021). Biostimulant-Treated Seedlings under Sustainable Agriculture: A Global Perspective Facing Climate Change. Agronomy.

[4] Murshed R., Lopez-Lauri F., Sallanon H. (2014). Effect of Salt Stress on Tomato Fruit Antioxidant Systems Depends on Fruit Development Stage. Physiol. Mol. Biol. Plants.

[5] Biswas S., Seal P., Majumder B., Biswas A.K. (2023). Efficacy of Seed Priming Strategies for Enhancing Salinity Tolerance in Plants: An Overview of the Progress and Achievements. Plant Stress.

[6] Habibi N., Aryan S., Amin M.W., Sanada A., Terada N., Koshio K. (2023). Potential Benefits of Seed Priming under Salt Stress Conditions on Physiological, and Biochemical Attributes of Micro-Tom Tomato Plants. Plants.

[7] Aloui H., Souguir M., Latique S., Hannachi C. (2014). Germination and Growth in Control and Primed Seeds of Pepper as Affected by Salt Stress. Cercet. Agron. Mold..

[8] Rahman M.M., Hossain M.A., Hossain K.F.B., Sikder M.T., Shammi M., Rasheduzzaman M., Hossain M.A., Alam A.M., Uddin M.K. (2018). Effects of NaCl-Salinity on Tomato (Lycopersicon Esculentum Mill.) Plants in a Pot Experiment. Open Agric..

[9] Habibi N., Tayobong R.R.P., Naoki P., Atsushi T., Kaihei S. (2024). Novel Insights into Seed Priming for Tomato Plants: Restoring Root Vitality in the Face of Salt Stress. Hortic. Environ. Biotechnol..

[10] Bacha H., Tekaya M., Drine S., Guasmi F., Touil L., Enneb H., Triki T., Cheour F., Ferchichi A. (2017). Impact of Salt Stress on Morpho-Physiological and Biochemical Parameters of *Solanum lycopersicum* Cv. Microtom Leaves. S. Afr. J. Bot..

[11] Kaveh H., Nemati H., Farsi M., Vatandoost Jartoodeh S. (2011). How Salinity Affect Germination and Emergence of Tomato Lines. J. Biol. Environ. Sci..

[12] Zhang Z., Chen Y., Wang C., Wang P., Tao F. (2017). Future Extreme Temperature and Its Impact on Rice Yield in China. Int. J. Climatol..

[13] Pérez-López U., Robredo A., Lacuesta M., Mena-Petite A., Muñoz-Rueda A. (2009). The Impact of Salt Stress on the Water Status of Barley Plants Is Partially Mitigated by Elevated CO2. Environ. Exp. Bot..

[14] Park H.J., Kim W.Y., Yun D.J. (2016). A New Insight of Salt Stress Signaling in Plant. Mol. Cells.

[15] Muhammad M., Waheed A., Wahab A., Majeed M., Nazim M., Liu Y.H., Li L., Li W.J. (2024). Soil Salinity and Drought Tolerance: An Evaluation of Plant Growth, Productivity, Microbial Diversity, and Amelioration Strategies. Plant Stress.

[16] Kesawat M.S., Satheesh N., Kherawat B.S., Kumar A., Kim H.U., Chung S.M., Kumar M. (2023). Regulation of Reactive Oxygen Species during Salt Stress in Plants and Their Crosstalk with Other Signaling Molecules—Current Perspectives and Future Directions. Plants.

[17] Fu H., Yang Y. (2023). How Plants Tolerate Salt Stress. Curr. Issues Mol. Biol..

[18] Alam M.S., Tester M., Fiene G., Mousa M.A.A. (2021). Early Growth Stage Characterization and the Biochemical Responses for Salinity Stress in Tomato. Plants.

[19] Ali M.M., Javed T., Mauro R.P., Shabbir R., Afzal I., Yousef A.F. (2020). Effect of Seed Priming with Potassium Nitrate on the Performance of Tomato. Agricultural.

[20] Biswas S., Rasal-Monir M., Islam M., Modak S., Humayun Kabir M. (2019). Induction of Salt Tolerance in Tomato Through Seed Priming. Plant.

[21] Hajer A.S., Malibari A.A., Al-Zahrani H.S., Almaghrabi O.A. (2006). Responses of Three Tomato Cultivars to Sea Water Salinity 1. Effect of Salinity on the Seedling Growth. Afr. J. Biotechnol..

[22] Kumar S., Singh T.B., Agnihotri R.K., Chaturvedi P. (2021). Comparative Effect of NaCl and PEG on Physiological and Biochemical Attributes during Vegetative Stage of Tomato CARAS. Res. Jr. Agril. Sci..

[23] Wang B., Wang J., Yang T., Wang J., Dai Q., Zhang F., Xi R., Yu Q., Li N. (2023). The Transcriptional Regulatory Network of Hormones and Genes under Salt Stress in Tomato Plants (*Solanum lycopersicum* L.). Front. Plant Sci..

[24] Nawaz A., Amjad M., Jahangir M.M., Khan S.M., Cui H., Hu J. (2012). Induction of Salt Tolerance in Tomato (Lycopersicon Esculentum Mill.) Seeds through Sand Priming. Aust. J. Crop Sci..

[25] Theerakulpisut P., Kanawapee N., Panwong B. (2016). Seed Priming Alleviated Salt Stress Effects on Rice Seedlings by Improving Na+/K+ and Maintaining Membrane Integrity. Int. J. Plant Biol..

[26] Garcia D., Zhao S., Arif S., Zhao Y., Ming L.C., Huang D. (2022). Seed Priming Technology as a Key Strategy to Increase Crop Plant Production under Adverse Environmental Conditions. J. Agric. Hortic. Res..

[27] Guo X., Zhi W., Feng Y., Zhou G., Zhu G. (2022). Seed Priming Improved Salt-Stressed Sorghum Growth by Enhancing Antioxidative Defense. PLoS ONE.

[28] Mirabi E., Hasanabadi M. (2012). Effect of Seed Priming on Some Characteristic of Seedling and Seed Vigor of Tomato (*Lycopersicun esculentum*). J. Adv. Lab. Res. Biol..

[29] Iqbal M., Ashraf M. (2007). Seed Treatment with Auxins Modulates Growth and Ion Partitioning in Salt-Stressed Wheat Plants. J. Integr. Plant Biol..

[30] Maggio A., Raimondi G., Martino A., Pascale S. (2007). De Salt Stress Response in Tomato beyond the Salinity Tolerance Threshold. Environ. Exp. Bot..

[31] Hameed A., Hussain S., Nisar F., Rasheed A., Shah S.Z. (2025). Seed Priming as an Effective Technique for Enhancing Salinity Tolerance in Plants: Mechanistic Insights and Prospects for Saline Agriculture with a Special Emphasis on Halophytes. Seeds.

[32] Chen K., Arora R. (2013). Priming Memory Invokes Seed Stress-Tolerance. Environ. Exp. Bot..

[33] Abdel Latef A.A., Tran L.S.P. (2016). Impacts of Priming with Silicon on the Growth and Tolerance of Maize Plants to Alkaline Stress. Front. Plant Sci..

[34] Dai L.Y., De Zhu H., De Yin K., Du J.D., Zhang Y.X. (2017). Seed Priming Mitigates the Effects of Saline-Alkali Stress in Soybean Seedlings. Chil. J. Agric. Res..

[35] Pradhan N., Prakash P., Tiwari S.K. (2015). Osmopriming of Tomato Genotypes with Polyethylene Glycol 6000 Induces Tolerance to Salinity Stress Osmopriming of Tomato Genotypes with Polyethylene Glycol 6000 Induces. Trends Biosci..

[36] Mitra D., Mondal R., Khoshru B., Shadangi S., Das Mohapatra P.K., Panneerselvam P. (2021). Rhizobacteria Mediated Seed Bio-Priming Triggers the Resistance and Plant Growth for Sustainable Crop Production. Curr. Res. Microb. Sci..

[37] Parra M., Albacete A., Martínez-Andújar C., Pérez-Alfocea F. (2007). Increasing Plant Vigour and Tomato Fruit Yield under Salinity by Inducing Plant Adaptation at the Earliest Seedling Stage. Environ. Exp. Bot..

[38] Kaya M.D., Ergin N., Harmancı P., Kulan E.G. (2024). Seed Priming as a Method of Preservation and Restoration of Sunflower Seeds. OCL—Oilseeds Fats Crop. Lipids.

[39] Ali S., Ullah S., Khan M.N., Khan W.M., Razak S.A., Wahab S., Hafeez A., Khan Bangash S.A., Poczai P. (2022). The Effects of Osmosis and Thermo-Priming on Salinity Stress Tolerance in *Vigna radiata* L.. Sustainability.

[40] Ishtiaq M., Mazhar M.W., Maqbool M., Hussain T., Hussain S.A., Casini R., Abd-ElGawad A.M., Elansary H.O. (2023). Seed Priming with the Selenium Nanoparticles Maintains the Redox Status in the Water Stressed Tomato Plants by Modulating the Antioxidant Defense Enzymes. Plants.

[41] Moradi L., Siosemardeh A. (2023). Combination of Seed Priming and Nutrient Foliar Application Improved Physiological Attributes, Grain Yield, and Biofortification of Rainfed Wheat. Front. Plant Sci..

[42] Abhinandan K., Skori L., Stanic M., Hickerson N.M.N., Jamshed M., Samuel M.A. (2018). Abiotic Stress Signaling in Wheat—An Inclusive Overview of Hormonal Interactions during Abiotic Stress Responses in Wheat. Front. Plant Sci..

[43] Jakab G., Cottier V., Toquin V., Rigoli G., Zimmerli L., Métraux J.-P., Mauch-Mani B. (2001). β-Aminobutyric Acid-Induced Resistance in Plants. Eur. J. Plant Pathol..

[44] Jisha K.C., Puthur J.T. (2016). Seed Priming with BABA (β-Amino Butyric Acid): A Cost-Effective Method of Abiotic Stress Tolerance in *Vigna radiata* (L.) Wilczek. Protoplasma.

[45] Wargent J.J., Pickup D.A., Paul N.D., Roberts M.R. (2013). Reduction of Photosynthetic Sensitivity in Response to Abiotic Stress in Tomato Is Mediated by a New Generation Plant Activator. BMC Plant Biol..

[46] Rhaman M.S., Imran S., Rauf F., Khatun M., Baskin C.C., Murata Y., Hasanuzzaman M. (2021). Seed Priming with Phytohormones: An Effective Approach for the Mitigation of Abiotic Stress. Plants.

[47] Lei C., Bagavathiannan M., Wang H., Sharpe S.M., Meng W., Yu J. (2021). Osmopriming with Polyethylene Glycol (PEG) for Abiotic Stress Tolerance in Germinating Crop Seeds: A Review. Agronomy.

[48] El Moukhtari A., Cabassa-Hourton C., Farissi M., Savouré A. (2020). How Does Proline Treatment Promote Salt Stress Tolerance During Crop Plant Development?. Front. Plant Sci..

[49] Wang R., Li C., Zeng L., Liu L., Xi J., Li J. (2024). Polyethylene Glycol Priming Enhances the Seed Germination and Seedling Growth of Scutellaria Baicalensis Georgi under Salt Stress. Plants.

[50] Nowicki M., Nowakowska M., Nowak K., Szczechura W., Kaminski P. (2025). Seed Priming and Abiotic Stress Tolerance in Carrot: Unraveling the Mechanisms of Improved Germination. PLoS ONE.

[51] Wang Y., Zhou E., Yao M., Xue D., Zhao N., Zhou Y., Li B., Wang K., Miao Y., Gu C. (2023). PEG-6000 Priming Improves Aged Soybean Seed Vigor via Carbon Metabolism, ROS Scavenging, Hormone Signaling, and Lignin Synthesis Regulation. Agronomy.

[52] Almakas A., Elrys A.S., Desoky E.-S.M., Al-Shuraym L.A., Alhag S.K., Alshaharni M.O., Alnadari F., NanNan Z., Farooq Z., El-Tarabily K.A. (2025). Enhancing Soybean Germination and Vigor under Water Stress: The Efficacy of Bio-Priming with Sodium Carboxymethyl Cellulose and Gum Arabic. Front. Plant Sci..

[53] Hamidian M., Kazemeini S.A., Movahhedi Dehnavi M., Ramezanian A., Mottaghi Jahromie M.R., Farsijani P., Iranshahi R., Mohebi P., Fereshteh Hekmat M., Hassani M. (2025). Individual and Combined Exogenous Application of Melatonin and Methyl Jasmonate Confer Salinity Stress Tolerance in Tomato by Enhancing Antioxidants Defense System. Sci. Hortic..

[54] Khan M., Hussain A., Yun B.-W., Mun B.-G. (2024). Melatonin: The Multifaceted Molecule in Plant Growth and Defense. Int. J. Mol. Sci..

[55] Samanta S., Roychoudhury A. (2023). Crosstalk of Melatonin with Major Phytohormones and Growth Regulators in Mediating Abiotic Stress Tolerance in Plants. S. Afr. J. Bot..

[56] Sairam R.K., Srivastava G.C., Agarwal S., Meena R.C. (2005). Differences in Antioxidant Activity in Response to Salinity Stress in Tolerant and Susceptible Wheat Genotypes. Biol. Plant..

[57] Zhu Z., Wei G., Li J., Qian Q., Yu J. (2004). Silicon Alleviates Salt Stress and Increases Antioxidant Enzymes Activity in Leaves of Salt-Stressed Cucumber (*Cucumis sativus* L.). Plant Sci..

[58] Gangur V., Gonipeta B., Kim E., Parvataneni S. (1994). Mechanism of Walnut Induced Anaphylaxis-like Shock Reaction in Mice (175.1). Physiol. Plant..

[59] Borsani O., Valpuesta V., Botella M.A. (2003). Developing Salt Tolerant Plants in a New Century: A Molecular Biology Approach. Plant Cell. Tissue Organ Cult..

[60] Mittova V., Guy M., Tal M., Volokita M. (2004). Salinity Up-Regulates the Antioxidative System in Root Mitochondria and Peroxisomes of the Wild Salt-Tolerant Tomato Species Lycopersicon Pennellii. J. Exp. Bot..

[61] Roșca M., Mihalache G., Stoleru V. (2023). Tomato Responses to Salinity Stress: From Morphological Traits to Genetic Changes. Front. Plant Sci..

[62] Yamaguchi K., Takahashi Y., Berberich T., Imai A., Miyazaki A., Takahashi T., Michael A., Kusano T. (2006). The Polyamine Spermine Protects against High Salt Stress in Arabidopsis Thaliana. FEBS Lett..

[63] Guo M., Wang X.-S., Guo H.-D., Bai S.-Y., Khan A., Wang X.-M., Gao Y.-M., Li J.-S. (2022). Tomato Salt Tolerance Mechanisms and Their Potential Applications for Fighting Salinity: A Review. Front. Plant Sci..

[64] Parvin K., Nahar K., Hasanuzzaman M., Bhuyan M.H.M.B., Mohsin S.M., Fujita M. (2020). Exogenous Vanillic Acid Enhances Salt Tolerance of Tomato: Insight into Plant Antioxidant Defense and Glyoxalase Systems. Plant Physiol. Biochem..

[65] Miransari M. (2014). Use of Microbes for the Alleviation of Soil Stresses.

[66] Raziq A., Wang Y., Mohi Ud Din A., Sun J., Shu S., Guo S. (2022). A Comprehensive Evaluation of Salt Tolerance in Tomato (Var. Ailsa Craig): Responses of Physiological and Transcriptional Changes in RBOH’s and ABA Biosynthesis and Signalling Genes. Int. J. Mol. Sci..

[67] Adhikari B., Olorunwa O.J., Horgan T.E., Wilson J., Barickman T.C., Li T., Bheemanahalli R. (2024). Seed Priming Attenuates the Impact of Salt Stress and Enhances Lettuce Yields. J. Agric. Food Res..

[68] Ahmad P., Abdel Latef A.A., Abd-Allah E.F., Hashem A., Sarwat M., Anjum N.A., Gucel S. (2016). Calcium and Potassium Supplementation Enhanced Growth, Osmolyte Secondary Metabolite Production, and Enzymatic Antioxidant Machinery in Cadmium-Exposed Chickpea (*Cicer arietinum* L.). Front. Plant Sci..

[69] Carvalho L.C., Vidigal P., Amâncio S. (2015). Oxidative Stress Homeostasis in Grapevine (*Vitis vinifera* L.). Front. Environ. Sci..

[70] Aydın A. (2024). The Growth, Leaf Antioxidant Enzymes and Amino Acid Content of Tomato as Affected by Grafting on Wild Tomato Rootstocks 1 (*S. pimpinellifolium* and *S. habrochaites*) Under Salt Stress. Sci. Hortic..

[71] Pérez-Labrada F., López-Vargas E.R., Ortega-Ortiz H., Cadenas-Pliego G., Benavides-Mendoza A., Juárez-Maldonado A. (2019). Responses of Tomato Plants under Saline Stress To. Plants.

[72] Borromeo I., Domenici F., Del Gallo M., Forni C. (2023). Role of Polyamines in the Response to Salt Stress of Tomato. Plants.

[73] Li S., Cui L., Zhang Y., Wang Y., Mao P. (2017). The Variation Tendency of Polyamines Forms and Components of Polyamine Metabolism in Zoysiagrass (*Zoysia japonica* Steud.) to Salt Stress with Exogenous Spermidine Application. Front. Physiol..

[74] Hu X., Zhang Y., Shi Y., Zhang Z., Zou Z., Zhang H., Zhao J. (2012). Effect of Exogenous Spermidine on Polyamine Content and Metabolism in Tomato Exposed to Salinity-Alkalinity Mixed Stress. Plant Physiol. Biochem..

[75] Jahan M.S., Shu S., Wang Y., Chen Z., He M., Tao M., Sun J., Guo S. (2019). Melatonin Alleviates Heat-Induced Damage of Tomato Seedlings by Balancing Redox Homeostasis and Modulating Polyamine and Nitric Oxide Biosynthesis. BMC Plant Biol..

[76] Marco F., Alcázar R., Tiburcio A.F., Carrasco P. (2011). Interactions between Polyamines and Abiotic Stress Pathway Responses Unraveled by Transcriptome Analysis of Polyamine Overproducers. Omi. A J. Integr. Biol..

[77] Raza M.A., Saeed A., Munir H., Ziaf K., Shakeel A., Saeed N., Munawar A., Rehman F. (2017). Screening of Tomato Genotypes for Salinity Tolerance Based on Early Growth Attributes and Leaf Inorganic Osmolytes. Arch. Agron. Soil Sci..

[78] Khalifa N.S. (2012). Protein Expression after NaCl Treatment in Two Tomato Cultivars Differing in Salt Tolerance. Acta Biol. Cracoviensia Ser. Bot..

[79] Zhao S., Zhang Q., Liu M., Zhou H., Ma C., Wang P. (2021). Regulation of Plant Responses to Salt Stress. Int. J. Mol. Sci..

[80] Michel B.E., Kaufmann M.R. (1973). The Osmotic Potential of Polyethylene Glycol 6000. Plant Physiol..

[81] Habibi N., Sediqui N., Terada N., Sanada A., Koshio K. (2021). Effects of Salinity on Growth, Physiological and Biochemical Responses of Tomato. J. ISSAAS.

[82] Han Y., Jiang J., Liu H., Ma Q., Xu W., Xu Y., Xu Z., Chong K. (2005). Overexpression of OsSIN, Encoding a Novel Small Protein, Causes Short Internodes in Oryza Sativa. Plant Sci..

[83] Gutteridge J.M.C. (1983). Superoxide Dismutases. Int. J. Radiat. Biol..

[84] Aebi H. (1984). Catalase in Vitro. Methods Enzymol..

[85] Dhindsa R.S., Plumb-Dhindsa P.L., Reid D.M. (1982). Leaf Senescence and Lipid Peroxidation: Effects of Some Phytohormones, and Scavengers of Free Radicals and Singlet Oxygen. Physiol. Plant..

[86] Nakano Y., Asada K. (1981). Hydrogen Peroxide Is Scavenged by Ascorbate-Specific Peroxidase in Spinach Chloroplasts. Plant Cell Physiol..

[87] Griffith O.W., Meister A. (1985). Origin and Turnover of Mitochondrial Glutathione. Proc. Natl. Acad. Sci. USA.

[88] Kranner I., Birtić S., Anderson K.M., Pritchard H.W. (2006). Glutathione Half-Cell Reduction Potential: A Universal Stress Marker and Modulator of Programmed Cell Death?. Free Radic. Biol. Med..

[89] Logan B.A., Demmig-Adams B., Adams W.W., Grace S.C. (1998). Antioxidants and Xanthophyll Cycle-Dependent Energy Dissipation in Cucurbita Pepo L. and Vinca Major L. Acclimated to Four Growth PPFDs in the Field. J. Exp. Bot..

[90] Walker-Simmons M. (1987). ABA Levels and Sensitivity in Developing Wheat Embryos of Sprouting Resistant and Susceptible Cultivars. Plant Physiol..

[91] Weiler E.W. (1980). Radioimmunoassays for the Differential and Direct Analysis of Free and Conjugated Abscisic Acid in Plant Extracts. Planta.

[92] Norman S.M., Poling S.M., Maier V.P. (1988). An Indirect Enzyme-Linked Immunosorbent Assay for (+)-Abscisic Acid in Citrus, Ricinus, and Xanthium Leaves. J. Agric. Food Chem..

[93] Gharsallah C., Fakhfakh H., Grubb D., Gorsane F. (2016). Effect of Salt Stress on Ion Concentration, Proline Content, Antioxidant Enzyme Activities and Gene Expression in Tomato Cultivars. AoB Plants.

[94] Zhang Y., Liang Y., Zhao X., Jin X., Hou L., Shi Y., Ahammed G.J. (2019). Silicon Compensates Phosphorus Deficit-Induced Growth Inhibition by Improving Photosynthetic Capacity, Antioxidant Potential, and Nutrient Homeostasis in Tomato. Agronomy.

[95] Hodges D.M., DeLong M.J., Forney F.C.P.K.R. (1999). Improving the Thiobarbituric Acid-Reactive-Substances Assay for Estimating Lipid Peroxidation in Plant Tissues Containing Anthocyanin and Other Interfering Compounds. Planta.

[96] Taulavuori E., Hellströ E.-K., Taulavuori K., Laine K. (2001). Comparison of Two Methods Used to Analyse Lipid Peroxidation from *Vaccinium Myrtillus* (L.) during Snow Removal, Reacclimation and Cold Acclimation. J. Experimetnal Bot..

[97] Oho K., Habibi N., Marie T., Silva B., Terada N., Sanada A., Shinohara T., Gemma H., Koshio K. (2022). Elucidation of physicochemical changes in fruit development of “sabara” jaboticaba (*Plinia cauliflora* (Mart.) Kausel). J. ISSAAS.

[98] Morgan J.A. (1984). Interaction of Water Supply and N in Wheat. Plant Physiol..

